# Oligomeric protein interference validates druggability of aspartate interconversion in *Plasmodium falciparum*

**DOI:** 10.1002/mbo3.779

**Published:** 2019-02-28

**Authors:** Fernando A. Batista, Soraya S. Bosch, Sabine Butzloff, Sergey Lunev, Kamila A. Meissner, Marleen Linzke, Atilio R. Romero, Chao Wang, Ingrid B. Müller, Alexander S. S. Dömling, Matthew R. Groves, Carsten Wrenger

**Affiliations:** ^1^ Department of Pharmacy, Structural Biology Unit, XB20 Drug Design University of Groningen Groningen The Netherlands; ^2^ Unit for Drug Discovery, Department of Parasitology, Institute of Biomedical Sciences University of São Paulo São Paulo Brazil; ^3^ LG Müller Bernhard Nocht Institute for Tropical Medicine Hamburg Germany

**Keywords:** drug target validation, oligomeric state, phenotypic mapping, *Plasmodium falciparum*, structural biology

## Abstract

The appearance of multi‐drug resistant strains of malaria poses a major challenge to human health and validated drug targets are urgently required. To define a protein's function in vivo and thereby validate it as a drug target, highly specific tools are required that modify protein function with minimal cross‐reactivity. While modern genetic approaches often offer the desired level of target specificity, applying these techniques is frequently challenging—particularly in the most dangerous malaria parasite, *Plasmodium falciparum*. Our hypothesis is that such challenges can be addressed by incorporating mutant proteins within oligomeric protein complexes of the target organism in vivo. In this manuscript, we provide data to support our hypothesis by demonstrating that recombinant expression of mutant proteins within *P. falciparum* leverages the native protein oligomeric state to influence protein function in vivo, thereby providing a rapid validation of potential drug targets. Our data show that interference with aspartate metabolism in vivo leads to a significant hindrance in parasite survival and strongly suggest that enzymes integral to aspartate metabolism are promising targets for the discovery of novel antimalarials.

## 1 INTRODUCTION

The parasite *Plasmodium falciparum* is responsible for the most lethal form of human malaria (World Health Organization, 2017). The spreading of *P. falciparum* in the human host depends on the availability of specific metabolites during its blood stage (Kirk & Saliba, 2007; Lindner, Meissner, Schettert, & Wrenger, 2013). The metabolism of these external nutrients represents a key‐step for parasite proliferation, and it is believed to be essential for its survivability. Although these metabolic steps would open new avenues for drug discovering targeting *P. falciparum*, the validation of these metabolic steps remains challenging due to limitations of applicability of probe techniques in *P. falciparum* and dependence upon reverse genetics (Meissner et al., 2016). This highlights the necessity of development of novel validation techniques capable of simplifying the validation of potential targets in *P. falciparum—*as well as other parasitic organisms. The examination of interaction surfaces between subunits of oligomeric proteins might offer a relatively straightforward alternative to this process (Meissner et al., 2016).

Protein oligomerization, the assembly of two or more copies of a single protein into one object, is a feature shared by all organisms and is present in more than 60% of all protein structures currently available within the Protein Data Bank (PDB; Hashimoto, Nishi, Bryant, & Panchenko, 2011). The biological importance, physicochemical properties, and evolutionary aspects of protein oligomerization have been recently summarized (Hashimoto & Panchenko, 2010; Hashimoto et al., 2011; Nishi, Hashimoto, Madej, & Panchenko, 2013). Furthermore, lower degree of evolutionary conservation of the oligomeric interfaces (Caffrey, Somaroo, Hughes, Mintseris, & Huang, 2004; Valdar & Thornton, 2001), as well as high specificity and binding affinity between the cognate partners, could successfully be utilized in drug target validation (Lunev et al., 2018). Based on these features, we hypothesized that the introduction of functionally incompetent forms of an enzyme into the native oligomeric assembly could be exploited in the analysis of biochemical pathways in vivo, particularly in cases where standard techniques (e.g., RNAi/knock in/out) have a low success rate.

The aspartate metabolism pathway within *P. falciparum* contains a number of oligomeric enzymes making it an ideal system to test our hypothesis that oligomeric self‐assembly can be used to modulate in vivo behavior. Aspartate interconversion is essential for nitrogen metabolism of all organisms. In *Plasmodium* species, it was also proposed to play a key role in de novo pyrimidine biosynthesis as well as energy metabolism (Wrenger et al., 2011). Plasmodial aspartate aminotransferase (*Pf*AspAT) and malate dehydrogenase (*Pf*MDH) catalyzes the reversible reaction from aspartate + 2‐oxoglutarate to oxaloacetate + glutamate and malate + NAD to oxaloacetate + NADH, respectively. The crystal structure of *Pf*AspAT has been previously solved (Wrenger et al., 2011). As reported, *Pf*AspAT is a homo‐dimer with a molecular weight of 48.42 kDa per monomer (Wrenger et al., 2011). Similarly to the previously described AspAT of *E. coli* (Jäger, Moser, Sauder, & Jansonius, 1994)*,* each subunit consists of three major domains: an N‐terminal arm (residues 1–14), a large coenzyme‐binding domain (residues 36–321) and a smaller domain (residues 15–36 and 322–404). The N‐terminal arm domain (residues 1–14) distal from either active site is thought to stabilize the *Pf*AspAT dimer and is necessary for activity, as truncated species lacking the N‐terminal extension showed significantly reduced activity while retaining dimeric structure (Wrenger et al., 2011). The two active sites of *Pf*AspAT are formed in a cleft between the big and small domains near the oligomeric interface and each active site pocket is composed of residues contributed from both subunits. Previous experiments where *Pf*AspAT was selectively inhibited in vitro using a polypeptide chain consisting of first 50 *Pf*AspAT amino acids (Wrenger et al., 2011) confirm the hypothesis that oligomeric interfaces show significantly higher sequence divergence amongst homologs and thus offer potential in specific interference with a target protein.

The structure *Pf*MDH has also been recently solved, and we have provided an insight into the role of oligomeric assembly in the regulation of *Pf*MDH activity (Lunev et al., 2018). *Pf*MDH possesses a tetrameric conformation where each monomer is comprised of 326 residues and is composed of two major domains: an N‐terminal cofactor‐binding domain containing a parallel structure of six beta‐sheets (Rossmann‐fold) and C‐terminal substrate‐binding domain. Our previous results suggested that a correctly formed tetrameric assembly of *Pf*MDH is essential for activity (Lunev et al., 2018). Indeed, the introduction of a tryptophan residue at one of the interfaces facilitating oligomerization (V190W) results in disruption of the tetramer, breaking it down into two dimers, and a significant reduction in activity. Co‐purification and western blot experiments with mixed lysates of recombinantly expressed wild‐type *Pf*MDH (Strep‐tagged) and *Pf*MDH‐V190W (His_6_‐tagged) mutant, with a predicted molecular mass of 35.28 and 36.74 kDa for each monomer, respectively, demonstrated that *Pf*MDH‐V190W was able to insert itself into a pre‐formed wild‐type *Pf*MDH assembly (Lunev et al., 2018). As shown by subsequent activity assays, the isolated wild‐type:V190W chimera possessed no detectable activity in either direction, while recombinant wild‐type *Pf*MDH displayed both reductive and oxidative activity. These data demonstrate that recombinant mutants can be used as specific modifiers of wild‐type *Pf*MDH activity in vitro, offering the potential to validate it as a drug target, without recourse to complex genetics or initial tool compounds that may display significant off‐target effects (Lunev et al., 2018). However, neither *Pf*AspAT nor *Pf*MDH has as yet been validated as a drug target in vivo.

In this study, structural information of the enzymes *Pf*MDH and *Pf*AspAT was used to generate mutants for use in in vivo protein interference experiments following two different approaches. In the first approach, we designed mutants that would incorporate within the native oligomer and disrupt the native oligomeric state, thereby inhibiting the function of the native assembly. In a second complementary approach, a mutant was designed to incorporate within the native oligomer and inhibit its function, without disruption to the native oligomeric state. In both approaches, the activity of the target enzymes was determined within the lysate of transgenic parasites, suggesting successful incorporation of the mutants within the targeted assemblies. The resulting data clearly indicate a significant dependence of the parasite on functional aspartate metabolism. Our data also provide proof‐of‐principle for protein interference assay (PIA) as a general approach to the use of oligomeric assemblies to obtain functional data in vivo.

## 2 RESULTS

### 2.1 The oligomeric interface of *Pf*AspAT shows higher sequence diversity than its active site

**Figure 1:**
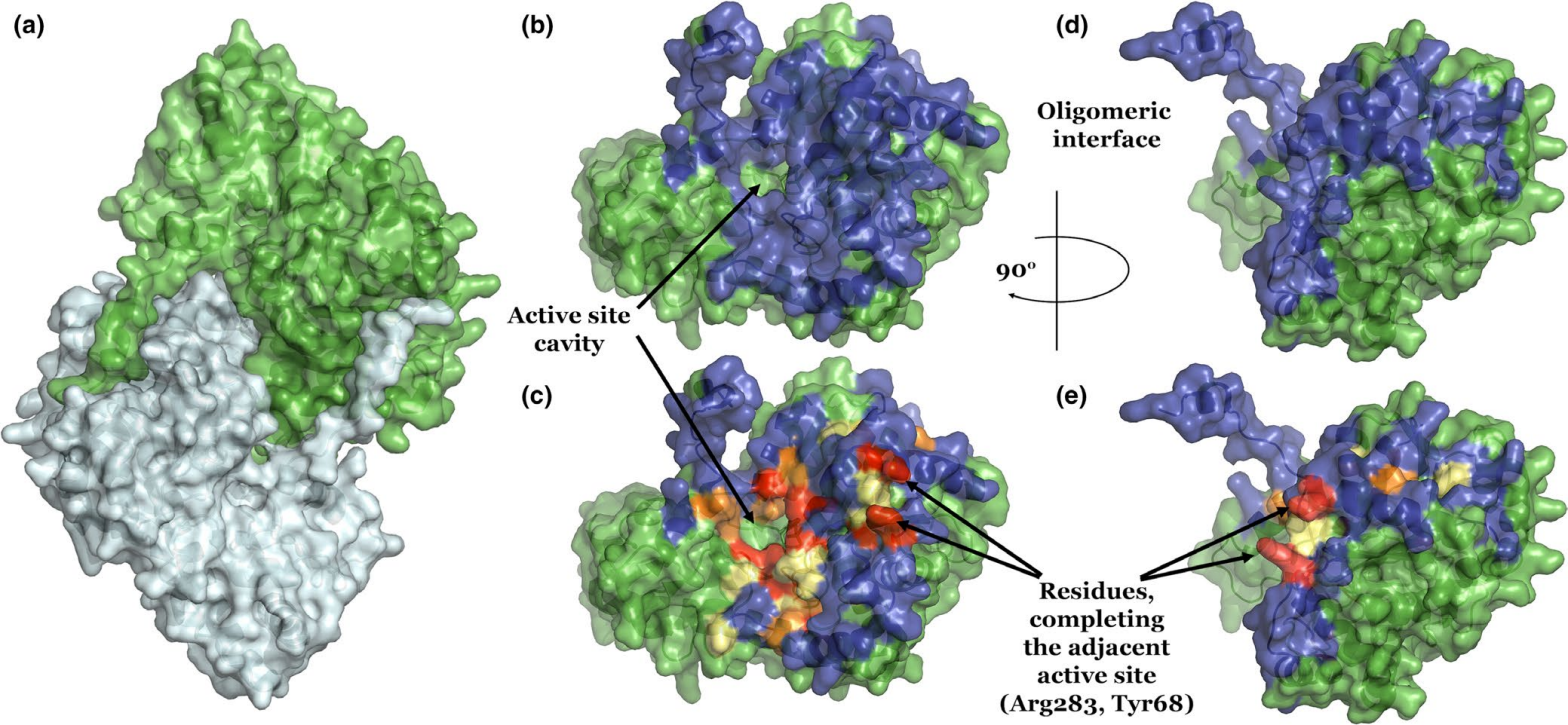
(a) *Pf*AspAT homodimer (3K7Y), where the N‐terminal arm domains are clearly visible. (b) and (d) (side view) Residues (blue) supporting oligomerization, as predicted by PISA (Krissinel & Henrick, 2007). (c) and (e) (side view) Evolutional diversity of the residues involved in the oligomeric contact: absolutely conserved (red), strongly conserved (orange) and slightly conserved (pale yellow). Sequence conservation amongst close homologs (identity over 28%) was analyzed using BLAST (Altschul et al., 1990). Figures were prepared using PyMol (Delano, 2018)

**Figure 2:**
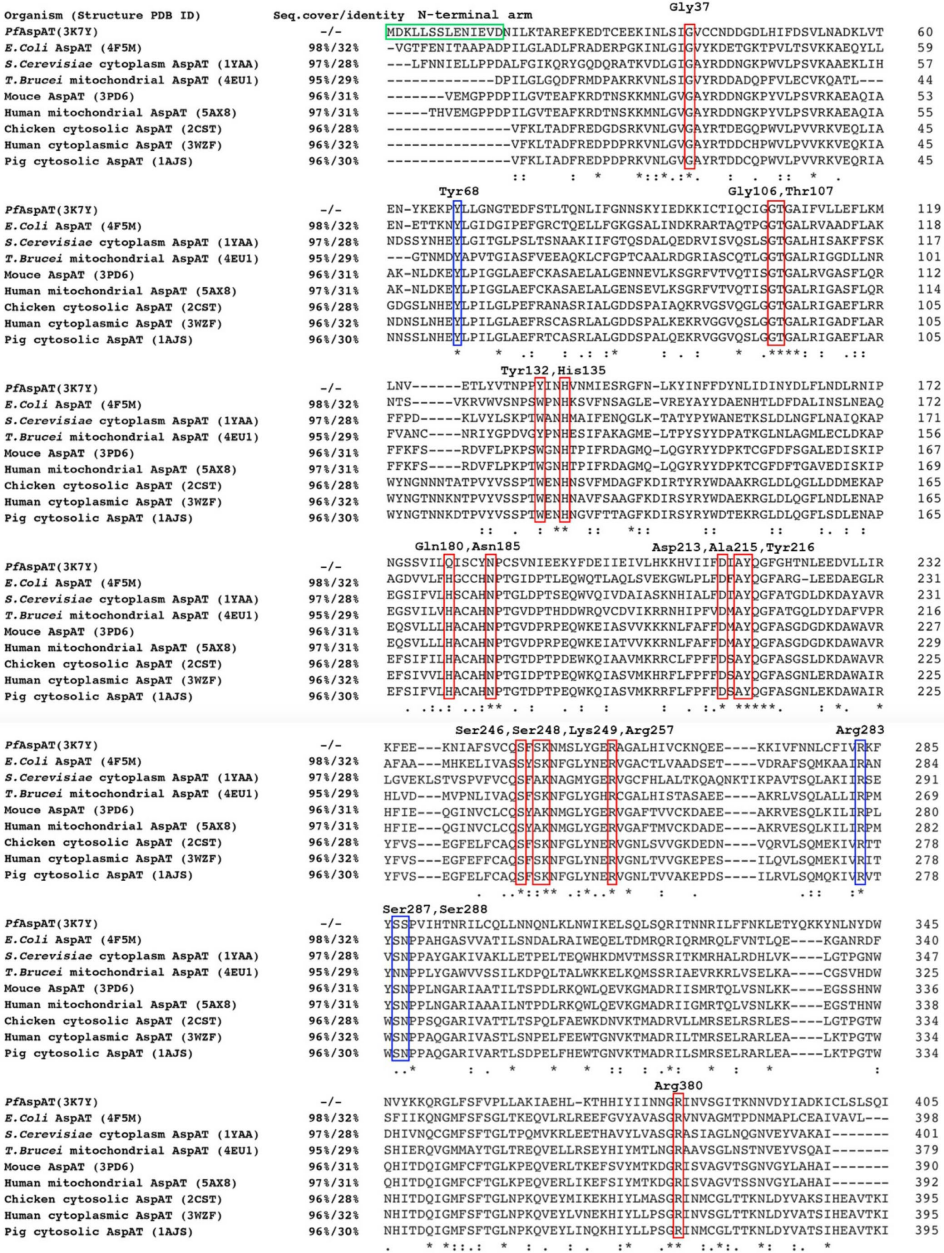
Sequence alignment of *Pf*AspAT against the closest homologs

BLAST (Altschul, Gish, Miller, Myers, & Lipman, 1990) analysis of the close homologs of *Pf*AspAT showed overall 38.7% sequence conservation, while the residues comprising the active site of *Pf*AspAT showed a sequence conservation of 100% (Table 1; Figure 1). *PISA (*Krissinel & Henrick, 2007) analysis of the structural assembly of *Pf*AspAT identified 98 residues involved in the inter‐oligomeric contact, showing sequence conservation of 34.7% (Table 1), where 10.2% accounts for the active site residues. As previously mentioned (Wrenger et al., 2011), the N‐terminal arm domain shows 100% sequence diversity (Figure 2). Only six residues involved in the contact with the N‐terminal arm domain (Asn277, Phe116, Ile263, Leu117, Val209, and Phe241) are somewhat conserved (Figures 1 and 2).

**Table 1:** Sequence conservation across the different oligomeric interfaces of *Pf*AspAT. Sequence conservation amongst close homologs (identity above 28%) was analyzed using BLAST (Altschul et al., 1990). Analysis of the residues supporting the oligomeric contact was performed using PISA (Krissinel & Henrick, 2007)

	*Pf*AspAT (PDB 3K7Y)
No. of residues	405
Conserved residues (% of total No.)	157 (38.7%)
Absolutely	49 (12.1%)
Strongly	66 (16.2%)
Weakly	42 (10.4%)
Active site residues (% of active site residues)	19
Absolutely	14 (73.7%)
Strongly	3 (15.8%)
Weakly	2 (10.5%)
Oligomeric interface	
Interface residues (% of interface residues)	98
Conserved residues	34 (34.7%)
Absolutely	11 (11.2%)
Strongly	12 (12.3%)
Weakly	11 (11.2)
Total ASA per monomer (Å^2^)	19,890 (100%)
Buried ASA (Å^2^)	2,436 (12.3%)

### 2.2 Point mutations of the key active site residues abolish catalytic activity of *Pf*AspAT in vitro while not disturbing the dimerization and overall fold

**Figure 3:**
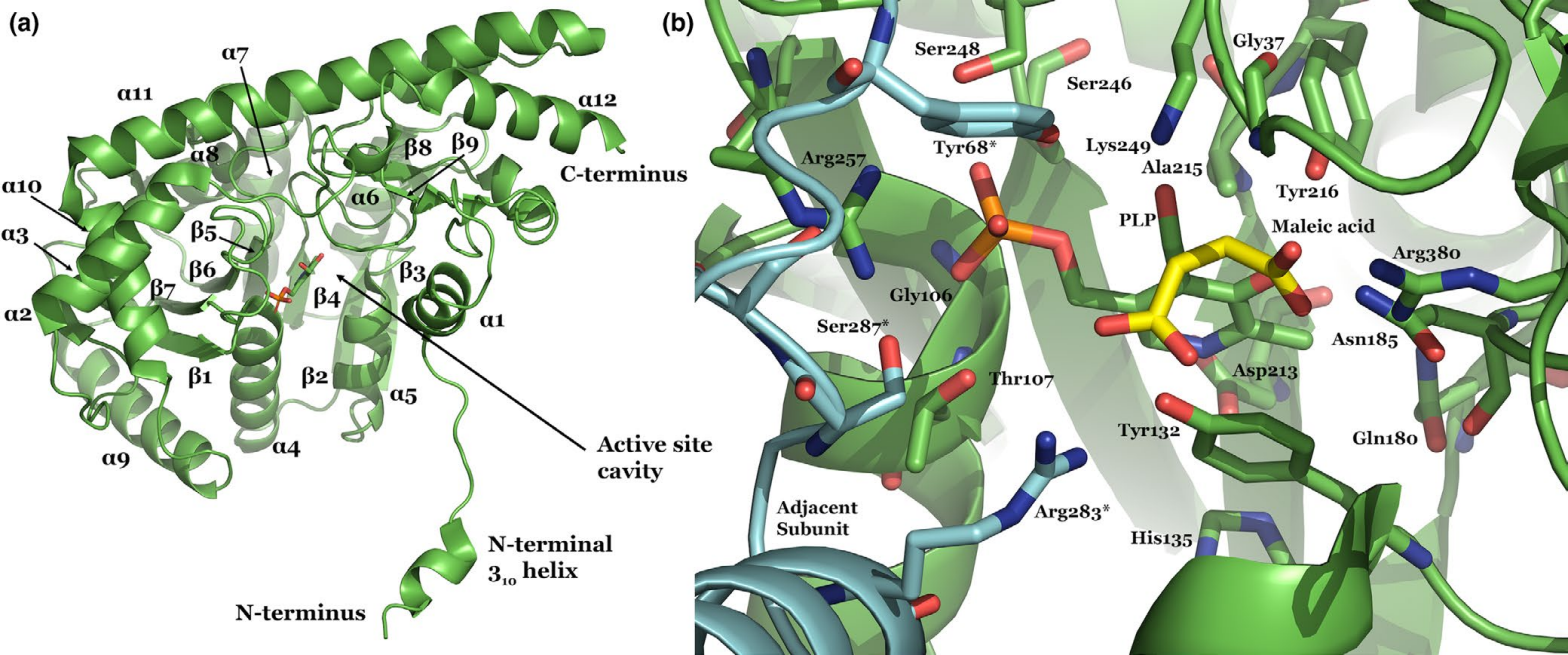
(a) Secondary structure of *Pf*AspAT (3K7Y). (b) *Pf*AspAT active site structure, in which the substrate analog maleic acid was modeled based on a homologous structure of chicken AspAT (2CST; Malashkevich et al., 1995) superimposed with *Pf*AspAT. Residues forming the active site cavity are shown in green; residues from the adjacent subunit complementing the active site are shown in cyan and labeled with asterisks. Figures were prepared using PyMol (Delano, 2018)

**Figure 4:**
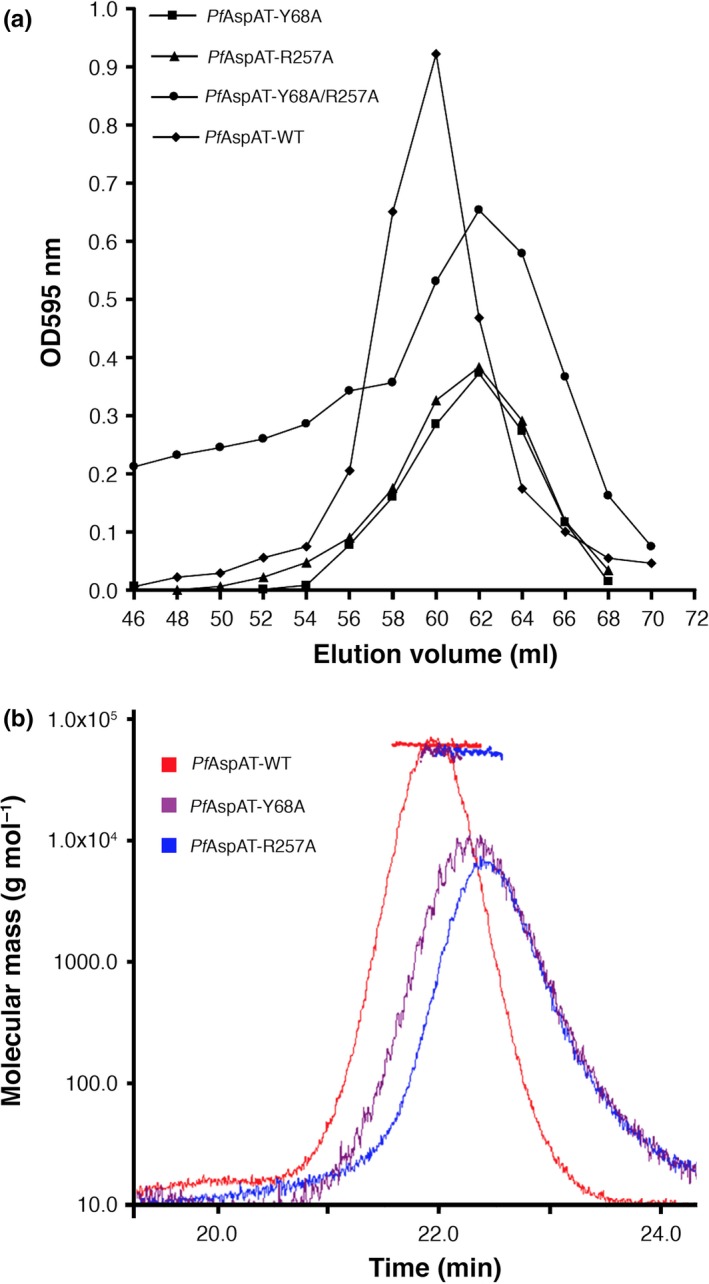
(a) Size‐exclusion chromatograms of *Pf*AspAT‐WT as well as its active site mutants. All samples were loaded onto S75 SEC column and eluted as single peaks with elution volumes of 60–62 ml. (b) SLS experiments of *Pf*AspAT‐WT and simple mutants, *Pf*AspAT‐Y68A and *Pf*AspAT‐R257A

**Figure 5:**
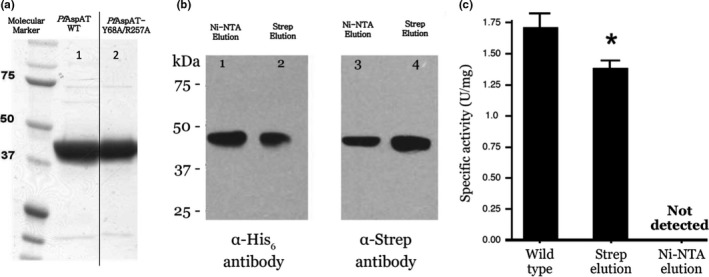
(a) SDS‐PAGE for Strep‐tagged wild‐type *Pf*AspAT (Lane 1) and His_6_ tagged *Pf*AspAT Y68A/R257A mutant (Lane 2) both purified from a co‐expression in *E. coli*. (b) western blot analysis shows that the wild‐type *Pf*AspAT (Strep‐tagged) and its inactive double mutant (Y68A/R257A, His_6_‐tagged) form a stable complex during co‐expression in *E. coli*. Lanes 1 and 3 contain samples purified using Ni‐affinity chromatography, while Lanes 2 and 4 contain samples purified using Strep tactin‐affinity chromatography. Lanes 1 and 2 and 3 and 4 were probed using a His_6_ antibody or Strep‐antibody respectively. The activities of the two eluates were then compared to that of the wild‐type. (c) Specific activity of the wild‐type *Pf*AspAT was similar to the previously reported value (Wrenger et al., 2011); Strep‐purified sample (selection for chimeras and wild‐type protein only) from co‐expression of Strep‐tagged WT and His_6_‐tagged *Pf*AspAT‐Y68A/R257A showed decreased activity, whereas no activity could be detected in the Ni‐NTA purified sample (selection for chimeras and inactive mutant only). GraphPad Prism 5.0 was used for one‐way ANOVA analysis

Based on the crystal structure of *Pf*AspAT (3K7Y; Wrenger et al., 2011), point mutations were designed in order to interfere with the catalytic activity of *Pf*AspAT. Arginine 257 and Tyrosine 68 of a single *Pf*AspAT monomer contribute to distinct catalytic sites and are both required for cofactor binding and catalytic functions (Figure 3). As previously described for *E.coli*, homologs of both residues (Arg266 and Tyr70; Jäger et al., 1994) are involved in hydrogen bonds to the phosphate group of the cofactor PLP. As a result, we hypothesized that mutations of these residues would significantly affect the catalytic activity of *Pf*AspAT. Furthermore, as Tyr68 and Arg257 belong to different subunits, the double mutation Y68A/R257A in a single monomer would affect both active sites of the dimer. The *Pf*AspAT‐Y68A/R257A mutant was recombinantly expressed and purified. Static light scattering (SLS) measurements confirmed that the introduction of both active site mutations did not impact the oligomeric assembly, as both wild type and mutant versions had a molecular weight of approx. 94 kDa, consistent with a dimeric assembly (Figure 4). The specific activity of the double mutant of *Pf*AspAT‐Y68A/R257A was measured, showing loss of activity (0.01 ± 0.0005 U/mg) compared to the wild type (1.71 ± 0.12 U/mg). This represents an approx. 170‐fold reduced catalytic activity of mutant (Figure 5). These data suggest that the introduction of two‐point mutations Y68A and R257A would have the desired inactivation effect on *Pf*AspAT while not affecting its folding or ability to form dimers.

### 2.3 Inactivated *Pf*AspAT mutant copies can be incorporated into the native assembly during recombinant expression in *E. coli*

Assuming that double mutation Y68A/R257A affects both active sites of one *Pf*AspAT dimer, we further hypothesized that formation of *Pf*AspAT wild‐type/mutant chimera would also affect both active sites and result in a significantly less active enzyme.

The *Pf*AspAT‐Y68A/R257A gene was sub‐cloned into the pBM1 vector using sequence‐specific primers (Appendix 1) and the resulting pBM1‐*Pf*AspAT‐Y68A/R257A plasmid encoded the full‐length *Pf*AspAT gene with both mutations and a His_6_‐tag fused at the C‐terminus. The wild‐type *Pf*AspAT with C‐terminal Strep‐tag was expressed according to a previously reported protocol (Jain, Jordanova, Müller, Wrenger, & Groves, 2010).

Recombinant co‐expression of both wild‐type *Pf*AspAT (Strep‐tagged) and *Pf*AspAT‐Y68A/R257A (His_6_‐tagged) mutant was performed in *E. coli* and the lysate from the co‐expression was purified by Strep tactin‐affinity and Ni‐affinity chromatography (Figure 5a). A western blot analysis of both eluates clearly demonstrated the presence of His_6_‐tagged double *Pf*AspAT mutant in a Strep‐purified sample (Figure 5b, lane 2) as well as the Strep‐tagged wild type in the His_6_‐purified sample (Figure 5b, lane 3). These results were interpreted as the formation of a dimer consisting of both wild type and mutant species of 2.4 *Pf*AspAT during co‐expression.

Further activity measurements indicated that Strep‐purified sample showed reduced activity compared to the wild type; while no activity could be detected from the His_6_‐purified sample (Figure 5c). These data indicate that single His_6_‐purification of the wild‐type:mutant *Pf*AspAT co‐expression product is able to isolate a chimeric oligomer consisting of the His_6_‐tagged *Pf*AspAT‐Y68A/R257A mutant and Strep‐tagged wild type. The lack of detectable activity of the purified chimera, confirms the hypothesis that a mutant copy (Y68A/R257A) can be introduced into the native *Pf*AspAT dimeric assembly through co‐expression resulting in an inactivated chimeric protein.

### 2.4 Introduction of PfAspAT and *Pf*MDH mutants results in a significant reduction in parasitaemia in aspartate‐limited culture media

**Figure 6:**
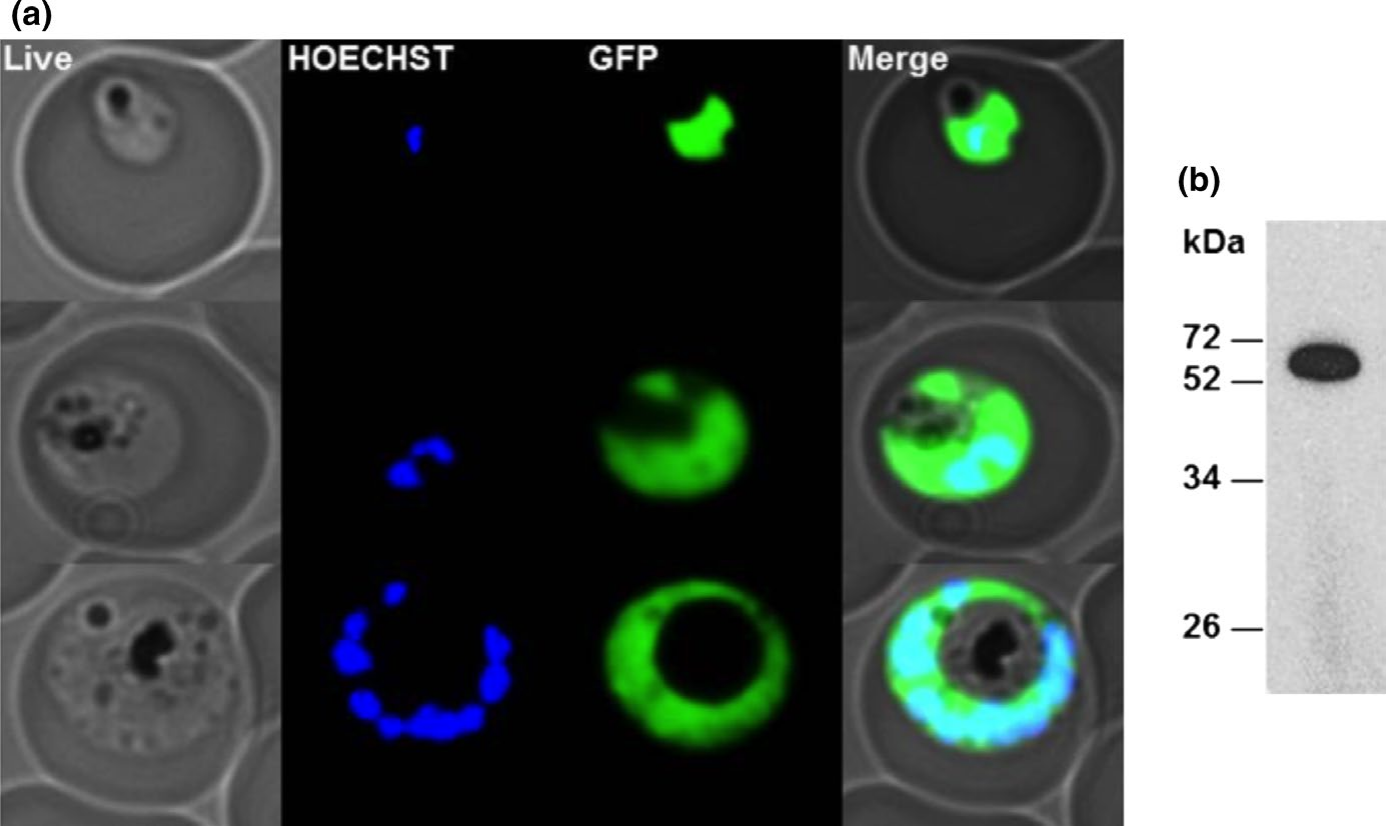
(a) Cytosolic localization of *Pf*MDH visualized inside the parasite by expression of a *Pf*MDH‐GFP chimera. (b) Protein expression of the transgenic cell line *Pf*MDH‐GFP chimera verified *via* western blot, the chimera has a molecular weight of 61.5 kDa

The cytosolic localization of *Pf*AspAT within the parasite has previously been reported (Wrenger et al., 2011). Similarly, the cytosolic localization of *Pf*MDH has been visualized by expression of a *Pf*MDH‐GFP chimera (Figure 6). In order to assess the effect of the presence of mutated *Pf*AspAT and *Pf*MDH in vivo, expression plasmids for *Pf*AspAT‐Y68A/R257A and *Pf*MDH‐V190W were created for transfection into *P. falciparum*. As the expression from the introduced plasmids is likely to result in an excess of the mutant protein with respect to the endogenous protein, it is not unreasonable to hypothesize that a significant proportion of the wild‐type oligomeric *Pf*MDH or *Pf*AspAT would contain at least a single copy of the mutant protein. Based upon our co‐expression and in vitro activity assays, the assembly of an endogenous *Pf*MDH with *Pf*MDH‐V190W or *Pf*AspAT with *Pf*AspAT‐Y68A/R257A would be expected to yield a dimeric assembly with no activity. Thus, the overexpression of *Pf*MDH‐V190W or *Pf*AspAT‐Y68A/R257A in vivo could be anticipated to result in inhibition of *Pf*MDH or *Pf*AspAT activity with little or no off‐target effects. As one of the major functions of these two proteins is to support the synthesis of aspartate this also allows an examination of the role of aspartate biosynthesis in blood‐stage cultures.

**Figure 7:**
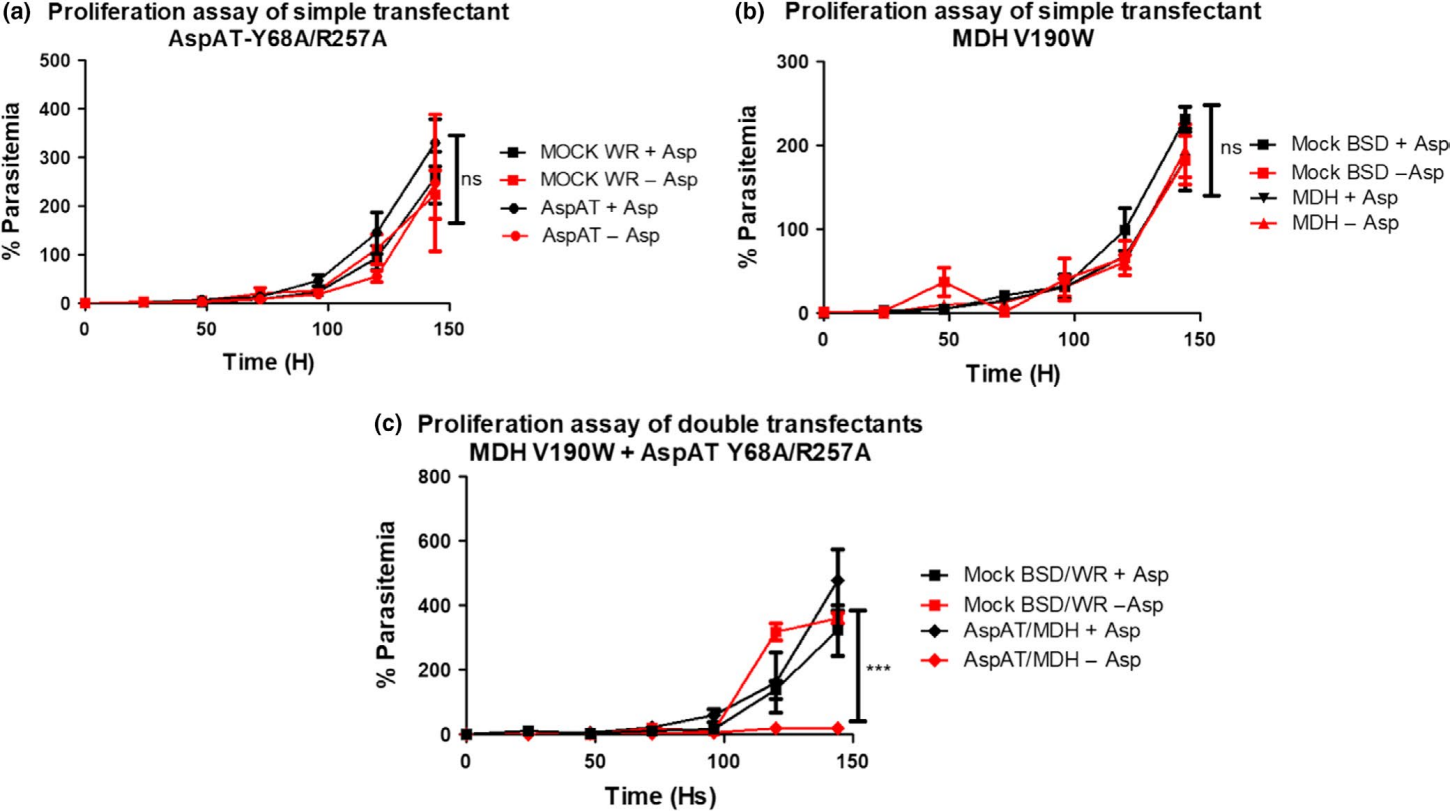
Proliferation curves of the transfected blood stage *Plasmodium falciparum* parasites. The values are given in percentage of parasitemia. Cultures were analyzed every 48 hr considering cumulative dilution steps. MOCK cell line was used as controls in all experiments. The experiments were performed with (+Asp) and without (‐Asp) the presence of aspartate in the media. (a) Introduction of *Pf*AspAT‐Y68A/R257A mutant alone did not cause any significant effect in the parasite viability. (b) Similarly, no significant effect of *Pf*MDH‐V190W introduction was observed. (c) While no effect of double transfection with *Pf*MDH‐V190W and *Pf*AspAT‐Y68A/R257A was observed in aspartate‐rich media, the parasite's viability was significantly hampered in the aspartate‐limited culture (p < o.oo1). Two‐way ANOVA analysis was performed using <>GraphPad Prism 5.0

**Figure 8:**
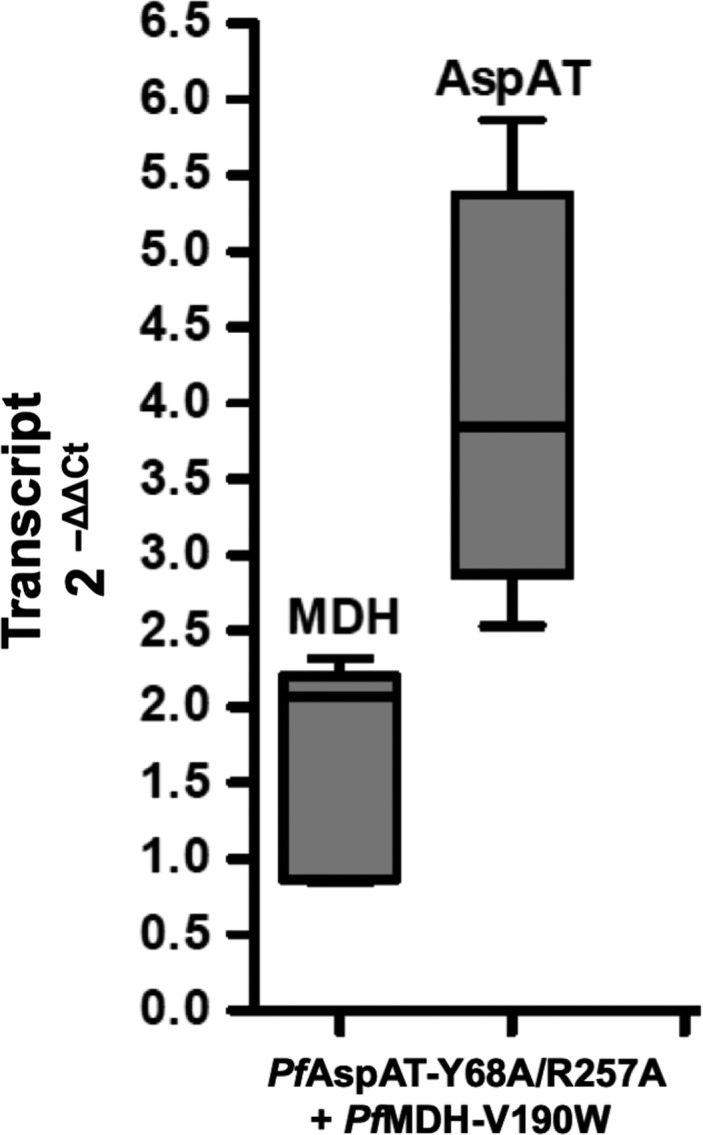
Transcription profile of AspAT‐Y68A/R257A + MDH‐V190W transgenic cell line. The analysis was done via the 2^−ΔΔCt^ method in triplicates and three independent experiments. The PCR was carried out with specific primers for both proteins. Aldolase was used as endogenous control. The values presented in the figure represent the relative amount of transcripts with the corresponding standard deviation

**Figure 9:**
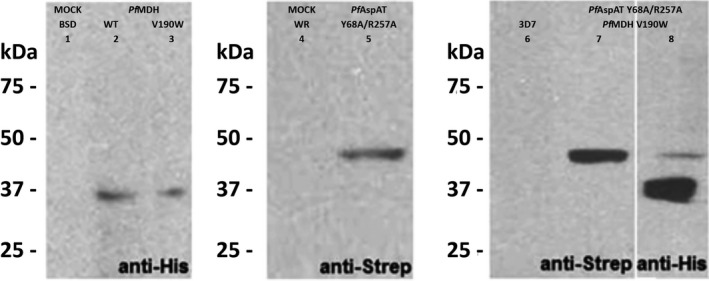
Protein expression of the transgenic cell lines *Pf*MDH‐WT (Lane 2), *Pf*MDH‐V190W (Lane 3), *Pf*AspAT‐Y68A/R257A (Lane 5) and the double transfect *Pf*AspAT‐Y68A/R257A with *Pf*MDH‐V190W (Lanes 7 and 8), verified via western blot. Control experiments with 3D7 parasites as well as the parasites transfected with MOCK plasmids are shown in Lanes 1, 4, and 6

For this reason, proliferation curves of the parasites were analyzed in different media. The growth of wild‐type parasites (control experiments using MOCK plasmids) was not significantly affected by the absence of aspartate in the culture media (Figure 7), providing a further demonstration that *P. falciparum* possesses a fully functional aspartate biosynthetic pathway. The introduction of *Pf*AspAT‐Y68A/R257A or *Pf*MDH‐V190W alone resulted in no detectable effects on blood‐stage parasite growth in aspartate rich or aspartate limiting media (Figure 7a,b). However, this demonstrates that there are no significant negative effects of introducing either the plasmids or the mutant proteins in vivo in terms of effects on parasite growth. While no effect is observed when both mutant proteins are introduced simultaneously in aspartate‐rich media, a significant effect (an almost 100‐fold reduction in parasitaemia) is seen on parasite proliferation in aspartate‐limiting cultures (Figure 7c). As neither mutant alone showed any negative effect on parasite growth in aspartate‐limited media, we interpret this data to mean that we have successfully inhibited both *Pf*MDH and *Pf*AspAT in vivo. As indicated above, our approach is unlikely to result in complete inhibition of either *Pf*AspAT or *Pf*MDH so the residual growth seen in the mutant‐inhibited cultures is most probably due to residual activity of uninhibited *Pf*AspAT and *Pf*MDH. Subsequently, to prove that the double‐transfect cell line expresses the transgenic proteins the overexpression of both transcripts was determined by qRT‐PCR. Compared to the wild‐type strain the *mdh‐* and *aspat‐*genes were 1.64‐fold ±0.32 and 4.10‐fold ±0.59 higher at the transcriptional level, respectively (Figure 8). Furthermore, the protein production within the transgenic parasites was visualized by western blotting from parasite lysate (Figure 9).

### 2.5 Activity measurements of *Pf*MDH and *Pf*AspAT in parasite lysates confirm the formation of the heterocomplexes

**Figure 10:**
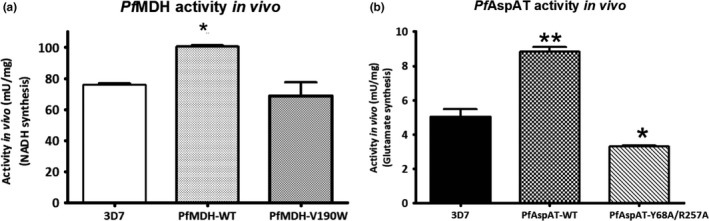
Specific activity values of *Pf*MDH (a) and (b) *Pf*AspAT, respectively, measured from *Plasmodium falciparum* cell lysates. The activity of the wild‐type MDH (a) was significantly higher compared to the V190W mutant (*p* < 0.05). The activity of AspAT‐WT (b) was significantly higher compared to both control (3D7) and double mutant (*p* < 0.05). Mutant *Pf*AspAT transfectant showed lower activity compared to the control (3D7; *p* < 0.05). The activity was measured in triplicates, in two independent experiments. GraphPad Prism 5.0 was used for one‐way ANOVA analysis

In order to confirm the activity profile of both enzymes in the respective transfected parasites, we performed specific activity assays using parasite extracts (Figure 10). As expected, proteins extracted from parasites transfected with plasmids encoding additional copies of wild‐type *Pf*MDH or *Pf*AspAT genes presented a statistically higher enzymatic activity (100.41 ± 1.05 and 8.83 ± 0.28 mU/mg, respectively) compared to non‐transfected parasites (3D7), confirming that both enzymes were not only being overexpressed (Figure 10a) but were also functional. The proteins extracted from parasites transfected with the mutant gene *Pf*MDH‐V190W showed a mild reduction in specific activity (68.78 ± 8.65 mU/mg) compared to the control (76.06 ± 0.68 mU/mg, Figure 10a). However, this difference was not statistically significant. The effect of the transfection with *Pf*AspAT‐Y68A/R257A mutant was more pronounced (3.32 ± 0.06 compared to the control of 5.03 ± 0.46 mU/mg, Figure 10b). These results confirm that the presence of mutant or extra wild‐type copies of proteins within the parasite could form complexes with the native enzymes and, as a consequence, decrease or increase the activity of the targeted oligomers.

## 3 DISCUSSION

In order to show the use of oligomeric surfaces in establishing protein interference studies in an in vivo setting, we have characterized the assembly surfaces of two plasmodial enzymes: aspartate aminotransferase (*Pf*AspAT) and malate dehydrogenase (*Pf*MDH), that are both localized in the cytosol of the parasite (Figure 6a; Wrenger et al., 2011). In order to validate the druggability of the targeted malate‐aspartate pathway, structure‐based mutagenesis experiments of the two involved genes were performed. We have recently demonstrated that a surface mutation in *Pf*MDH, where valine 190 was mutated into tryptophan, disrupted the A‐C interface as anticipated, causing significant activity loss (Lunev et al., 2018). Our previous data suggested that the introduction of the V190W mutation at the oligomeric interface (A‐C) not only caused the splitting of the tetramer into a pair of dimers but it also made the re‐formation of the tetramer highly unlikely due to the introduction of molecular clashes. It was also previously reported that the mutated *Pf*MDH‐V190W was able to incorporate into the native *Pf*MDH‐WT assembly in vitro, disturbing the native oligomeric state of the target protein as well as inhibiting its activity (Lunev et al., 2018). In this work, we also demonstrate that the hypothesized *Pf*MDH chimeras are formed inside the parasite in vivo, through measurement of *Pf*MDH activity in the lysate of transgenic parasites.

We performed similar experiments with *Pf*AspAT. In contrast with *Pf*MDH where the oligomeric state is disturbed, in our *Pf*AspAT mutant, the native oligomeric state is maintained. Incorporation of mutant *Pf*AspAT showed that the enzyme's native oligomeric assembly could also be utilized in order to target the wild‐type protein and show an effect on activity in vitro (Figure 5c) and in vivo (Figure 10). The inactive *Pf*AspAT‐Y68A/R257A mutant was shown to be able to incorporate into native *Pf*AspAT‐WT dimeric assembly during recombinant co‐expression, as confirmed by western blot (Figure 5b), resulting in complete loss of activity (Figure 5c). The mutated *Pf*AspAT protein was then expressed in *P. falciparum* blood‐stage cultures, and we have demonstrated that the hypothesized *Pf*AspAT chimeras are formed inside the parasite in vivo, through measurement of *Pf*AspAT activity in the lysate of transgenic parasites. While no effect is seen in aspartate‐rich media, the introduction of both mutant proteins in aspartate‐limited media results in a clear phenotype (Figure 7), without recourse to complex genetic manipulations. We have termed this approach the oligomeric protein interference assay (PIA; Meissner et al., 2016).

Our data show that oligomeric surfaces can be used to specifically inhibit protein activity in vivo*,* especially in cases when opportunities for genetic manipulation are limited. The introduction of *Pf*AspAT‐Y68A/R257A or *Pf*MDH‐V190W alone did not result in any significant effect on parasite proliferation in blood‐stage cultures (Figure 7a,b). Nonetheless, we believe that these findings demonstrate that the expression of mutant proteins in cultured parasites has overall no negative effects on parasite growth. This also indicates that, while the expression levels of the mutant proteins are significantly higher (up to fourfold) than their endogenous counterparts, they are likely not overexpressed at a level that would induce metabolic stress on *P. falciparum* through depletion of amino acids. As the mutant proteins introduced are designed and demonstrated to be inactive, it is also highly unlikely that we have significantly altered the metabolic balance within the parasite. In addition, our experiments utilized well‐characterized expression plasmids to introduce the mutant proteins and numerous previous publications have not reported significant effects of these plasmids on parasite growth (Butzloff, 2013; Knöckel et al., 2012; Meissner et al., 2016; Müller et al., 2009). The lack of negative effect on the proliferation of culture parasites is supported by the measurements of specific activity of both enzymes in lysates of parasites transfected with the mutant genes, which show that these parasites retain a partial activity of both enzymes (Figure 10a,b). Additionally, continuous expression of the inserted mutant reduces the risk of possible degradation of the mutant protein by cellular proteases before it reaches the intended targets. Further, our mutations were designed to result in inactive proteins (confirmed by our in vitro activity assays (Lunev et al., 2018), Figure 3c). In fact, the activity assay in lysates of parasites supports not only the hypothesis that the introduction of mutated proteins will cause a decrease in the activity, but also indicates that the formation of heterocomplexes occurs in vivo, thereby influencing the function of the native protein.

Finally, in contrast to current small molecule inhibition approaches, no limitations regarding drug or compound delivery to the cytosol are encountered as the mutant proteins are expressed directly within the parasite. Based on this analysis, we believe that the introduction of inactive proteins specific for oligomeric targets represents a minimally perturbing method to specifically inhibit metabolic pathways of interest in the human malaria parasite *P. falciparum* that will have a minimal off‐target effect, and, in this manner, offers a possible tool to be used in the validation of target candidates.

While the insertion of the individual mutations of *Pf*MDH or *Pf*AspAT results in no negative effect on parasitemia, the transfection of parasites with both plasmids results in a significant reduction in parasite proliferation in aspartate‐limited media (Figure 7c). This is a clear change in phenotypic behavior that has been generated without recourse to complex genetic approaches. These data strongly suggest that while the correct function of either *Pf*MDH or *Pf*AspAT is sufficient to support parasite proliferation during the blood stage, simultaneous inhibition of both results in a significant reduction in parasite growth. While our data support the concept of oligomeric protein interference assays for cytosolic proteins, in principle the use of the native targeting sequence will also allow the approach to be used for proteins present in other cellular compartments (mitochondrial, apicoplast, membrane inserted, etc.).

**Figure 11:**
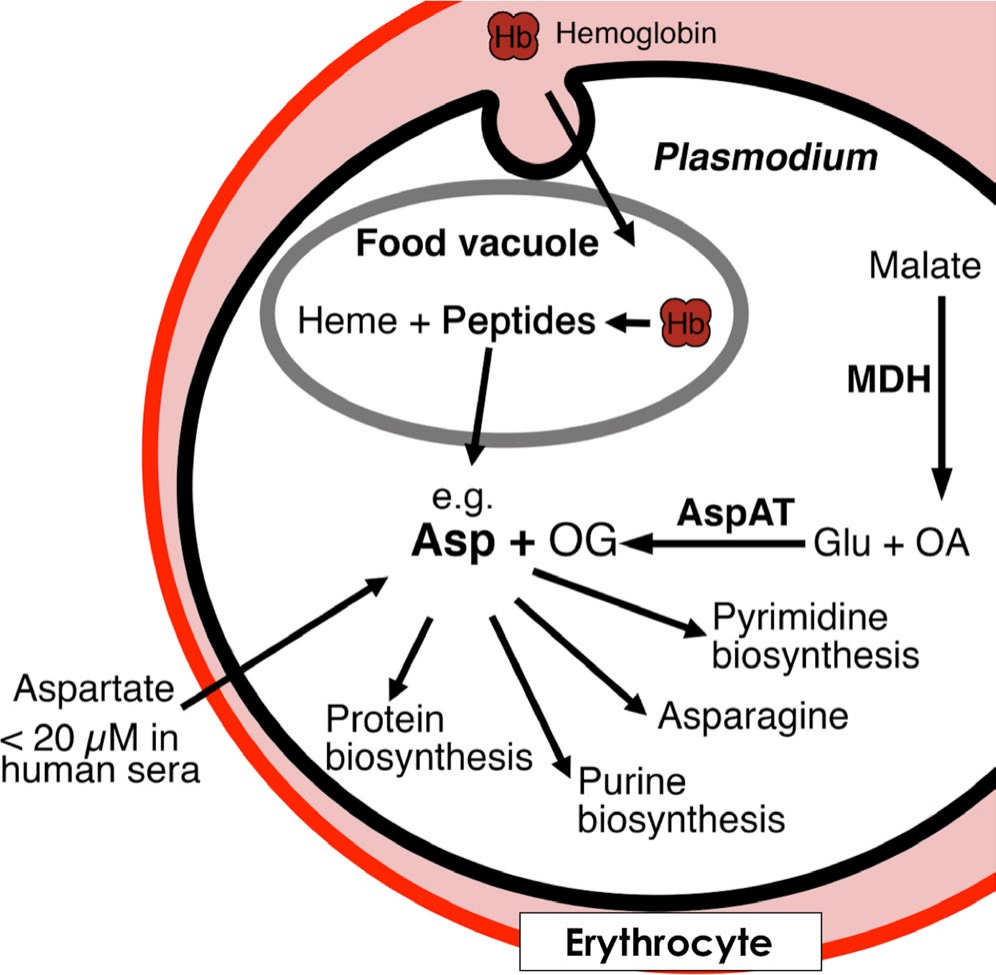
Importance of the aspartate metabolism in *Plasmodium falciparum*. Our experiments showed that inhibition of the de‐novo aspartate biosynthesis via *Pf*MDH and *Pf*AspAT is a viable target for future antimalarial drug design

A number of recent manuscripts have focused on elucidating the role of the mitochondria of the malarial parasite (Ke et al., 2015; Nixon et al., 2013). Amongst the pathways supported by the mitochondria are those involved in the biosynthesis of aspartate. While aspartate is essential in the formation of new proteins, it is also a key precursor in pyrimidine biosynthesis (Cassera, Zhang, Hazleton, & Schramm, 2011; Hyde, 2007), another promising pathway for drug discovery, as confirmed by validation of dihydroorotate dehydrogenase (DHODH) as a drug target (Vyas & Ghate, 2011). During proliferation, the malaria parasite catabolizes hemoglobin as an amino acid source. Although aspartate is available in hemoglobin, the host cell protein does not sufficiently suit the need of *P. falciparum* for the rapid proliferation within blood stage as demonstrated by the presented growth experiments of blood‐stage cultures in aspartate‐limited media. Thus, aspartate biosynthesis or uptake is highly likely to be a key element in supporting the rapid proliferation of *P. falciparum* in human red blood cells. It has long been known that aspartate is the least common of all the amino acids available within the human serum, with recent measurements suggesting that the concentration of L‐aspartate in human sera is <20 µM (Psychogios et al., 2011). This strongly suggests that biosynthetic pathways would be the main source of the aspartate required by *P. falciparum* in the human host (Figure 11).

The significant drop in proliferation upon oligomeric‐based inhibition of both *Pf*MDH and *Pf*AspAT in aspartate‐limited cell culture media suggest that aspartate biosynthesis in the malarial parasite depends upon the function of both of these enzymes and validates this metabolic pathway as drug target in *P. falciparum*. This is in accord with recent data that suggest the products of glycolysis (both pre‐ and post‐mitochondrial) are used in biosynthesis in the malarial parasite (Ke et al., 2015). In another recent study (Zhang et al., 2018), mutagenic index scores (MISs) calculations have predicted *Pf*MDH as an essential enzyme. In contrast, the PIA experiments revealed no effect on the proliferation of MDH mutant overexpressing parasites. However, this down‐regulation of intra‐cellular MDH activity will not warrant a total “knock‐down” of the plasmodial MDH due to the presence of residual endogenous wild‐type MDH. On the other hand, the MIS approach suggested *Pf*AspAT as non‐essential, corroborating with our data, which shows no effect on the knock‐down of *Pf*AspAT alone although an intra‐cellular simultaneous over‐expression of *Pf*AspAT and *Pf*MDH mutated proteins clearly causing a severe growth defect. While the simultaneous inhibition of two enzymes may be highly challenging for the development of novel antimalarials, our data strongly suggest that future drug targets to treat malaria infection may be found within downstream components of the aspartate metabolism pathway (Figure 11). Taken together, our data also show that oligomeric surfaces offer a highly promising opportunity to specifically influence protein behavior in vivo and offer a novel avenue in the validation of pathways for downstream drug development, particularly in the field of infectious diseases.

## 4 MATERIAL AND METHODS

### 4.1 Expression and purification of recombinant *Pf*AspAT

The purification of *Pf*AspAT has been previously reported (Jain et al., 2010). Wild‐type *Pf*AspAT open reading frame (ORF) was cloned into pASK‐IBA3 expression plasmid with additional C‐terminal His_6_‐tag to facilitate purification via Ni‐NTA chromatography. The generated pASK‐IBA3‐*Pf*AspAT plasmid was transformed into the commercially available *E. coli* expression strain BLR (DE3; Novagen) for expression. After expression, the cells were lysed using sonication and centrifuged to separate the lysate. Soluble His‐tagged *Pf*AspAT was purified using Ni‐NTA agarose (Quiagen) according to the manufacturer's recommendations. *Pf*AspAT was further purified via size‐exclusion chromatography using HiLoad 16/60 Superdex S75 column (GE Healthcare).

### 4.2 Site‐directed mutagenesis

The single mutants *Pf*AspAT‐Y68A and *Pf*AspAT‐R257A, and the double mutant *Pf*AspAT‐Y68A/R257A were generated via site‐directed mutagenesis using specific oligonucleotides containing the altered codons (Appendix 1) and the pASK‐IBA3‐*Pf*AspAT plasmid (Jain et al., 2010) as a template. All constructs were verified by Sanger sequencing.

### 4.3 Determination of oligomeric state

The analysis of the oligomeric state of recombinant WT *Pf*AspAT and mutants was performed according to the previously described protocol (Wrenger et al., 2011). Briefly, *Pf*AspAT‐WT, *Pf*AspAT‐Y68A, *Pf*AspAT‐R257A, and *Pf*AspAT‐Y68A/R257A samples were applied onto Superdex S75 10/300 (GE Healthcare) size exclusion column. The wild‐type *Pf*AspAT sample eluted as a single peak with at approx. 60 ml, while all three mutant samples eluted somewhat later at approx. 62 ml (Figure 8). Further SLS analysis (MiniDAWN TREOS [Wyatt]) confirmed the dimeric state of the wild‐type *Pf*AspAT as well as mutants, with an approximate weight of 97.5 kDa (including the purification tags).

### 4.4 Recombinant co‐expression and co‐purification of *Pf*AspAT‐WT and *Pf*AspAT‐Y68A/R257A double mutant

The wild‐type *Pf*AspAT ORF was re‐cloned into pASK‐IBA3 and the resulting plasmid‐encoded full‐length *Pf*AspAT‐WT with C‐terminal Strep‐tag. The His_6_‐tagged *Pf*AspAT‐Y68A/R257A double mutant was sub‐cloned into pACYC184 vector (NEB) containing the expression cassette of pJC40 to allow co‐expression of the WT and mutant version in *E. coli*. The co‐expression of Strep‐tagged *Pf*AspAT‐WT and His_6_‐tagged *Pf*AspAT‐Y68A/R257A was performed using co‐transformed BLR (DE3) competent cells induced with both IPTG and AHT. The co‐purification was performed via the Strep‐tactin as well as via Ni‐NTA agarose (Qiagen). The co‐purified proteins were visualized by western blot using a monoclonal Strep‐tag II antibody (IBA) or anti‐His antibody (Pierce, USA) and a secondary anti‐mouse horseradish peroxidase‐labeled goat antibody (Bio‐Rad, Germany).

### 4.5 In vitro activity assays

The specific activity of the double mutant of *Pf*AspAT‐Y68A/R257A was measured following the same procedure as previously reported (Wrenger et al., 2011). The effect of incorporation of mutant His_6_‐tagged *Pf*AspAT‐Y68A/R257A into the native Strep‐tagged wild‐type assembly was also analyzed using the samples of the co‐expression experiments (described above).

### 4.6 Cloning and transfection of *Pf*MDH V190W and *Pf*AspAT Y68A, R257A

In order to obtain transgenic parasites, the ORFs of WT‐*Pf*AspAT and *Pf*AspAT‐Y68A/R257A were amplified via PCR using sequence‐specific primers (Appendix 1) and subsequently cloned into pARL 1a‐ with the hDHFR (human dihydrofolate reductase) resistance cassette (Wrenger & Müller, 2004). The resulting plasmids encoded for the full‐length WT‐*Pf*AspAT and *Pf*AspAT‐Y68A/R257A mutant with an additional C‐terminal Strep‐tag followed by the stop‐codon before the GFP gene encoded on pARL 1a‐. Similarly, WT‐*Pf*MDH and the *Pf*MDH‐V190W mutant with C‐terminal His_6_‐tag (cloning procedure described in (Lunev et al., 2018)) were cloned into pARL 1a‐ with BSD (Blasticidin S) resistance cassette in order to facilitate double transfection (Knöckel et al., 2012).

In order to determine WR992010 and Blasticidin S drug‐selection effects, two MOCK plasmids were generated (pARL‐MOCK‐hDHFR, pARL‐MOCK‐BSD) as described in (Knöckel et al., 2012). All constructs were confirmed by automatic sequencing (Sanger) before transfection into *P. falciparum*. The transfection of the resulting constructs pARL‐*Pf*AspAT‐Y68A/R257A‐Strep‐hDHFR, pARL‐*Pf*MDH‐V190W‐his‐BSD, pARL‐MOCK‐hDHFR and pARL‐MOCK‐BSD was performed into ring stage *P. falciparum* 3D7 as described in (Knöckel et al., 2012; Müller et al., 2010). The co‐transfected cell lines were generated using the stabilized parasites previously transfected with pARL‐*Pf*AspAT‐Y68A/R257A‐Strep‐hDHFR or pARL‐MOCK‐hDHFR, which were then electroporated with pARL‐*Pf*MDH‐V190W‐his‐BSD or pARL‐MOCK‐BSD plasmids, respectively. The selection of transgenic parasites was performed using 5 nM of WR99210 and 1 μg/ml Blasticidin S. Transfected parasites were maintained in continuous culture using the conditions of Trager and Jensen modified as described in (Das et al., 2005).

### 4.7 qRT and western blot

An asynchronous culture of transgenic 3D7 parasites was isolated via saponin lysis. The total RNA of these parasites was extracted using TRIZOL following the manufacturer's instruction. The cDNA synthesis was performed as described in (Butzloff, 2013; Chan et al., 2013; Knöckel et al., 2012; Müller et al., 2010). The quantitative real‐time PCR was performed using specific primers (Appendix 1), that identify products from 150–190 bp. Briefly, 2 μl of each primer (5 pmol/μl) were used together with 9 μl of 2.5× Real Master Mix SYBR (20×; 5Prime), 6 μl RNAse‐free dH_2_O and 1 μl cDNA (50 ng/μl). The reaction was performed in a thermocycler (Corbett Cycler) with the following program: 2 min 95°C followed by 35 cycles of 95°C for 15 s, 49°C for 20 s and 69°C for 20 s, and a final step of 95°C for 2 min. Normalization and calibration were performed using the aldolase gene (Salanti et al., 2003) and the respective MOCK cell line like (Chan et al., 2013). The data were analyzed using the Corbett Rotor‐Gene 6.1.81 software and the 2^−ΔΔCt^ method (Livak & Schmittgen, 2001).

The protein expression of the transgenic cell lines was verified via western blot analysis as described in (Knöckel et al., 2012). Briefly, isolated parasites were resuspended in 5x SDS‐PAGE sample buffer, boiled for 5 min at 95°C and centrifuged for 5 min at 14,000 ×*g*. The supernatant was separated by 10% SDS‐PAGE and transferred on a nitrocellulose membrane (Bio‐Rad). The expressed proteins were detected via their Strep‐ or His_6_‐tag by using a monoclonal anti Strep‐ or anti‐His antibody (1:5,000 dilution (IBA; Pierce) and a secondary anti‐mouse HRP‐labeled antibody (1:10,000 dilution; Pierce) and visualized on X‐ray films using the SuperSignal West Pico detection system (Thermo Scientific).

### 4.8 Activity assays from parasites lysate

In order to analyze the specific activity of *Pf*AspAT and *Pf*MDH, we performed activity assays in parasites lysates. For this analysis, cultures of transgenic cell lines pARL‐*Pf*AspAT‐WT, pARL‐*Pf*AspAT‐Y68A/R257A, pARL‐*Pf*MDH‐WT, and pARL‐*Pf*MDH‐V190W, as well as the WT 3D7 culture used as a control, were isolated via saponin lysis.

The specific activity of *Pf*MDH was measured with the Malate Dehydrogenase Assay Kit (Sigma Aldrich). The reaction was carried out at 37°C in a final volume of 150 µl, according to the manufacturer's protocol. The absorbance was monitored at 450 nm.

The specific activity of *Pf*AspAT was measured with the Aspartate Aminotransferase (AST) Activity Assay Kit (Sigma Aldrich). The reaction was carried out at 37°C in a final volume of 100 µl, according to the manufacturer's protocol. The absorbance was also monitored at 450 nm. The amount of total protein in the lysates was quantified by Bradford assay (Bradford, 1976).

### 4.9 Proliferation assays

In order to analyze the long‐term influence of the overexpressing cell lines pARL‐*Pf*AspAT‐Y68A/R257A‐Strep‐hDHFR, pARL‐*Pf*MDH‐V190W‐his‐BSD and the double transgenic cell line pARL‐*Pf*AspAT‐Y68A/R257A‐strep‐hDHFR + pARL‐*Pf*MDH‐V190W‐his‐BSD in comparison with their respective MOCK line, parasites growth was monitored over several days. The parasites were synchronized using sorbitol and a starting parasitaemia of 1% of ring‐stage iRBC (infected red blood cells) was adjusted. Giemsa‐stained thin smears were analyzed daily and the parasitaemia was determined by light microscopy in percentage of iRBC to total RBC. Cultures with more than 8% of iRBC were diluted and cumulative parasitaemias were calculated as described in (Knöckel et al., 2012). Triple repetition of the proliferation assay was performed and the growth curves were generated with GraphPad Prism 4.0. The slope of the respective curves was calculated through an exponential equation (Müller et al., 2009).

### 4.10 Localization of *Pf*MDH

The ORF of *Pf*MDH‐WT with no stop‐codon at C‐terminus was amplified by PCR and subsequently cloned into pARL 1a‐ using KpnI/AvrII restriction enzymes (Appendix 1). The resulting plasmid encoded for the wild‐type *Pf*MDH fused in front of the GFP gene and was transfected into *P. falciparum* 3D7 parasites as described above. The localization of the MDH‐GFP chimera was analyzed via live cell fluorescent microscopy (Müller et al., 2010) using an Axio Imager M2 microscope (Zeiss) equipped with an AxioCam HRC digital camera (Zeiss). In order to visualize the nucleus, the parasites were incubated with 10 µg/ml HOECHST 33342 dye (Invitrogen). The images were analyzed with the AxioVision 4.8 software.

## CONFLICT OF INTEREST

The authors declare no conflict of interest.

## AUTHORS CONTRIBUTION

FAB, SL, SB, CW, and ARR and performed protein expression and purification experiments. SB, ML, IBM, and KAM performed mutagenesis experiments. FAB, SSB, and SB performed proliferation/activity assays. FAB, ARR, and SL performed SLS analysis. ASSD, MRG, and CW designed and coordinated the research. FAB, SSB, CW, and MRG wrote the manuscript, with contribution from all authors. All authors reviewed the results and approved the final version of the manuscript.

## ETHICS STATEMENT

None required.

## Data Availability

All data supporting this study are provided in full in the results section of this paper.

## APPENDIX 1

Primer sequences used in the study. The recognition sites for restriction enzymes used (specified in the primer name) are highlighted in bold. Mutations sites are underlined

**Table:**

Primer	Sequence
*Pf*AspAT cloning for recombinant expression (pASK‐IBA3, His_6_‐tag)	
IBA3‐AspAT‐s (BsaI, His6)	5′‐GCGCGC**GGTCTC**CAATGGATAAGTTATTAAGCAGCTTAG‐3′
IBA3‐AspAT‐as (BsaI, His6)	5′‐GCGCGC**GGTCTC**AGCGCTTTAATGATGATGATGATGATGGCCCTGAAA ATAAAGATTCTCTATTTGACTTAGCGAAAGACAAATTTTGTCGG‐3′
*Pf*AspAT cloning for recombinant expression (pASK‐IBA3, Strep‐tag)	
IBA3‐AspAT‐s (BsaI, Strep)	5′‐GCGC**GGTCTC**CAATGGATAAGTTATTAAGCAGCTTAG‐3′
IBA3‐AspAT‐as (BsaI, Strep)	5′‐GCGC**GGTCTC**TGCGCTTATTTGACTTAGCGAAAGAC‐3′
*Pf*AspAT Site‐directed mutagenesis primers (pASK‐IBA3)	
AspAT‐R257A‐s	5′‐ATGTCGCTTTATGGAGAAGCAGCAGGTGCTCTTCATATTG‐3′
AspAT‐R257A‐as	5′‐CAATATGAAGAGCACCTGCTGCTTCTCCATAAAGCGACAT‐3′
AspAT‐Y68A‐s	5′‐GAAAATTATAAAGAGAAACCAGCATTGTTAGGTAACGGTACAGAA‐3′
AspAT‐Y68A‐as	5′‐TTCTGTACCGTTACCTAACAATGCTGGTTTCTCTTTATAATTTTC‐3′
*Pf*AspAT sub‐cloning primers for recombinant co‐expression in *E. coli* (pACYC184)	
pACYC184‐AspT‐s (NdeI)	5′‐AGAG**CATATG**GATAAGTTATTAAGCAGCTTAG‐3′
pACYC184‐AspT‐as (XmaI)	5′‐ATAT**CCCGGG**TCATATTTGACTTAGCGAAAGA‐3′
*Pf*AspAT double mutant sub‐cloning primers for in vivo overexpression (pARL 1a‐)	
pARL‐AspAT‐s (KpnI)	5′‐GAGA**GGTACC**ATGGATAAGTTATTAAGCAGCTTAG‐3′
pARL‐AspAT‐Strep‐as (XhoI)	5′‐GCGC**CTCGAG**TTATTATTTTTCGAACTGCGGGTGGC‐3′
*Pf*AspAT qPCR primers	
qPCR‐AspAT‐s	5′‐CGCCTTTTCGGTATGTCAATCC‐3′
qPCR‐AspAT‐as	5′‐AGTTGGCATAGAATACGATTCGT‐3′
*Pf*MDH double mutant sub‐cloning primers for in vivo overexpression (pARL 1a‐)	
pARL‐MDH‐s (SmaI)	5′‐GAGA**CCCGGG**ATGGTACCAGCTCGAGCCTAGGATGACTAAAATT GCCTTAATAGG‐3′
pARL‐MDH‐His‐as (XhoI)	5′‐GAGA**CTCGAG**TTATTAATGATGATGATGATGATG‐3′
*Pf*MDH qPCR primers	
qPCR‐MDH‐s	5′‐CCCAGCTGCTGCTATTACAA‐3′
qPCR‐MDH‐as	5′‐CTGGATGTGCCCCCTTATTA‐3′
*Pf*Aldolase qPCR control gene	
qPCR‐*Pf*Aldolase‐s	5′‐TGTACCACCAGCCTTACCAG‐3′
qPCR‐*Pf*Aldolase‐as	5′‐TTCCTTGCCATGTGTTCAAT‐3′
